# Exosome-mediated transfer of CD44 from high-metastatic ovarian cancer cells promotes migration and invasion of low-metastatic ovarian cancer cells

**DOI:** 10.1186/s13048-021-00776-2

**Published:** 2021-02-24

**Authors:** Xiameng Shen, Conghui Wang, Huihui Zhu, Yaping Wang, Xinyu Wang, Xiaodong Cheng, Wanzhong Ge, Weiguo Lu

**Affiliations:** 1 Department of Gynecologic Oncology, Women’s Hospital School of Medicine Zhejiang University, No. 1 Xueshi Road, Hangzhou, 310006 China; 2 Women’s Reproductive Health Research Laboratory of Zhejiang Province, Women’s Hospital School of Medicine Zhejiang University, Hangzhou, China; 3 Zhejiang University Cancer Center, Hangzhou, China

**Keywords:** Exosome, Ovarian cancer, Heterogeneity, Metastasis

## Abstract

**Objective:**

To investigate the detailed roles and mechanisms of tumor-derived exosomes in progression and metastasis of ovarian cancer in vitro.

**Methods:**

Exosomes were isolated by differential centrifugation method; the morphology, size and biological markers of exosomes were separately defined by transmission electron microscopy, nanoS90 and Western blotting; Trans-well chambers assay was used to assess the ability of migration and invasion of recipient cells uptaking the exosomes from HO8910PM cells. The downstream molecule was screened by mass spectrometry.CD44 was identified by western blotting and the function of CD44 was identified by trans-well chambers assay and CCK8 assay.

**Results:**

Exosomes derived from HO8910PM cells could be transferred to HO8910 cells and promote cell migration and invasion in the recipient cells of ovarian cancer. And CD44 could be transferred to the HO8910 cells through exosomes from HO8910PM cells and influence the migration and invasion ability of HO8910 cells.

**Conclusion:**

The more aggressive subpopulation can transfer a metastatic phenotype to the less one via secreting exosomes within a heterogeneous tumor. CD44 may be a potential therapeutic approach for ovarian cancer.

## Introduction

Ovarian cancer is the most lethal type in gynecological neoplasms. In the USA, around 22,440 women were diagnosed as ovarian cancer and 14,070 women died from the disease in 2018. The overall 5-year survival rate of ovarian cancer patients was 47% according to cancer statistics, but dropped to 29% in advanced stage patients, gathered over the period from 2006 to 2012 [42]. The high mortality related to ovarian cancer is due to its aggressive behavior and metastatic potential, but the underlying mechanism remains unclear.

Cancers, including ovarian cancer, frequently display substantial intra-tumor heterogeneity in virtually all distinguishable phenotypic features, such as cellular morphology, gene expression (including the expression of cell surface markers and growth factors and hormonal receptors), metabolism, motility, angiogenic, proliferative, immunogenic, and metastatic potential [9, 11, 17, 28]. However, all those heterogeneous cells tend to homogenous in the similiar microenvironment. For example, all cancer cells eventually present similar aggressive potential via intercellular interactions without outside intervention. And perhaps there might be a network of biological interactions among the distinct clones. Understanding the mechanism would help to improve the treatment strategies for cancer.

Exosomes are small extracellular vesicles (EVs), ranging from 30 to 150 nm in size. They are produced by all cells and present in all body fluids [6, 20]. In the past, releasing exosomes was considered as a form of disposing of cellular wastes [30]. Today, exosomes are emerging as excellently equipped vehicles for information transfer between cells [5, 47]. The protein and microRNA content of exosomes has been implicated in various intracellular processes that mediate oncogenesis, tumor spread, and drug resistance. Tumor cells actively produce, release, and utilize exosomes to promote tumor growth and convey molecular and genetic messages from tumor cells to normal or other abnormal cells residing at close or distant sites. However, it remains unknown whether exosomes are involved in the process of homogenization of heterogeneous tumor cells, and the content of exosomes which may work still needs further research.

In this study, we presented the evidence that exosomes derived from high metastatic ovarian cells can be transferred to low metastatic ovarian cancer cells and promote the migration and invasion of recipient cells. Furthermore, we intended to find the molecules in exosomes through MS analysis and identified CD44 in transferred exosomes was a mediator in promoting metastatic behavior during this process. Our findings may provide a novel approach for ovarian cancer therapeutics.

## Materials and methods

### Cell culture

The human epithelial ovarian cancer cell lines, HO8910 and HO8910PM, were acquired from the Women’s Hospital, School of Medicine, Zhejiang University, where they were tested and authenticated. They were not cultured continuously for more than 3 months. Adherent HO8910 and HO8910PM cells were cultured in Roswell Park Memorial Institute (RPMI)-1640 medium (BI, Kibbutz Beit-Haemek, Israel), supplemented with 10% fetal bovine serum (FBS) (Invitrogen, New York, USA) and 100 U/mL penicillin, and 100 μg/mL streptomycin, maintained at 37 °C in 5% CO_2_ and detached using trypsin/EDTA solution.

### Plasmids transfection

CD44 variant1 was cloned into the pEX-2 vector, X-treme GENE HP DNA Transfection Reagent (Roche, Basel, Switzerland) was used for transient transfection (Roche, Basel, Switzerland) following the manufacturer’s protocols. For G418 (Sigma-Aldrich, St. Louis, USA) selection, cells were transfected with plasmid for 24 h and treated with 500 μg/mL G418 for 14 days.

### Migration assay

Trans-well plates (24-well, 8-mm pore size; Costar, Cambridge, MA, USA) were used to conduct the migration assay. The lower chamber of the trans-well plate was filled with 500uL of 1640 medium containing 10% FBS. Cells (2 × 10^5^) suspended in 1640 medium without FBS were added to the upper chamber. The plate was incubated at 37 °C with 5% CO_2_ in air for 24 h. At the end of the assay, after removing non-migrating cells by scraping from the top of the filter, each filter was fixed in methanol, stained with 0.5% crystal violet (Sigma-Aldrich, St. Louis, USA) and cells were observed by a light microscope at 400 × magnification. Each assay was performed in triplicate; migrating cells were counted in at least five high power fields per well.

### Matrigel invasion assay

In vitro invasion assay was evaluated in trans-well chambers. Briefly, cells (3 × 10^5^) suspended in serum-free RPMI 1640 medium with 8 μm pore filter coated with 1:12 matrigel (BD Biosciences, San Jose, CA, USA) and exposed to medium supplemented with 10% of FBS for 24 h at 37 °C in 5% CO_2_. At the end of the assay, after removing non-invading cells by scraping from the top of the filter, each filter was fixed in methanol, stained with 0.5% crystal violet, and cells were observed by a light microscope at 400× magnification. Each assay was performed in triplicate, invading cells were counted in at least five high power fields per well.

### Cell viability assays

Cells were seeded (4000 cells per well) in 96-well plates, after adhering to the plates for 24 h, 48 h, 72 h, and 96 h, the cell viability was determined with Cell Counting Kit-8 (Dojindo, Japan), following the manufacturer’s protocols.

### Isolation of exosomes

When reached 50–60% confluency, cells were washed with PBS twice and incubated with RPMI-1640 containing 10% exosome-depleted FBS (prepared by 16 h overnight ultracentrifugation at 120,000×g at 4 °C) for 48 h. Exosomes were isolated from the conditioned medium by ultracentrifugation. In brief, conditioned medium was centrifuged at 300×g for 10 min, then at 2000×g for 10 min at 4 °C to remove cells, lastly, at 10000×g for 30 min at 4 °C to remove cell debris. Exosomes were pelleted by ultracentrifugation at 100,000×g for 90 min. They were resuspended in PBS and collected by ultracentrifugation again at 100,000×g for 90 min. The concentration of the exosomes were quantified by BCA (Beyotime, Shanghai, China) and exosomes were ready for cell treatment.

### Transmission electron microscopy

Exosome pellets, dissolved in PBS buffer were dropped in a carbon-coated copper grid and then stained with 1% uranyl acetate. The samples were observed using a JEM-1230 transmission electron microscope (JEOL, Japan).

### Measurement of particle size and concentration distribution

Nanoparticles in exosome suspensions were analyzed using a Litesizer 500 (Anton Paar GmbH, Austria).

### Labeling and internalization of exosomes

The purified exosomes were labeled with a PKH67 green fluorescent labeling kit (Sigma-Aldrich, St. Louis, USA), according to the manufacturer’s instructions. Briefly, the washed exosome pellets were resuspended in 250 μl of Dilution C, PKH67 (2 μl) was diluted in 250 mL Dilution, then they were mixed gently for 3 min, and an equivalent volume of 0.1% BSA was added to bind the excess PKH67. PKH67-labeled exosomes were extracted through ultracentrifugation and the pellets were suspend in PBS. Finally, the recipient cells were co-cultured with the PKH67-labeled exosomes for 4 h and 24 h.Actin filaments were stained with Palloidin (red stain) (Beyotime, Shanghai, China). DNA were stained with DAPI (blue stain) (Abcam, Cambridge, MA, USA). Images were taken using a fluorescence microscope to confirm the presence of exosomes within the cells and analyzed by ImageJ software.

### LC-MS/MS analysis

All peptide samples were dried by vacuum centrifugation and resuspended in 2% ACN and 0.1% FA. Then, peptides weres separated by nanoLC-MS/MS using an UltiMate 3000 RSLCnano system (Thermo Fisher, Waltham, MA, USA) and analyzed by Q Exactive HF-X (Thermo Fisher, Waltham, MA, USA). Solvent A is 2% ACN, 0.1% FA and solvent B is 98% ACN, 0.1% FA. Gradient elution was performed at 32 °C using linear gradients of 120 min at a flow rate of 400 nl/min: 1–4 min with 3% (v/v) of s B, 4–6 min from 3 to 5% (v/v) B, 6–70 min from 5 to 15% (v/v) B, 70–90 min from 15 to 30% (v/v) B, 90–100 min from 30to 80% (v/v) B, 100–110 min with 80% (v/v) B, 110–120 min with 3% (v/v) A. MS spectra were acquired using 120,000 resolution with a mass range of 300-1500 m/z and an AGC target of 3E6. MS2 spectra were acquired using 45,000 resolution and HCD fragmentation performed with collision energy approximately 32% NCE. In addition, we used a method of data-dependent top 20 on MS2 with an AGC target of 2E5. Isolation window was 1.0 m/z, charge exclusion was ≤2 and ≥ 7, and dynamic exclusion was set to 30 s. The fixed first mass was 100 m/z, peptide match was preferred, and exclude isotopes was on.

### Peptide-protein identification and quantification

MaxQuant (version 1.6.2.10) was used for protein identification and quantification (Cox and Mann, 2008; Cox et al.,2011). The human UniProtKB database (October 2018) and HBV-b/−c database (October 2018) were utilized as the search database, while the automatic reverse database and known contaminants were used for decoy search. Variable modifications included oxidation (M) (+ 15.99491 Da), acetyl (protein N-term) (+ 42.01056 Da), and GlyGly(K)_10plex_TMT (+ 343.20586 Da). Carbamidomethyl (C) (+ 57.02146 Da) was set as the fixed modification, and the max number of modifications per peptide was set to 5. Trypsin was set as the specificity of digestion, and max missed cleavage sites was 2. We used 20 ppm in the first search ion tolerance and 4.5 ppm in the main search ion tolerance. Both peptide and protein identification were performed at FDR < 1%. Except for the above-mentioned, the default parameters of MaxQuant were adopted.

### RNA extraction and qPCR

RNA extraction kit (TaKaRa, Beijing, China) was used to extract RNA from cultured cells. The cDNA was generated using the PrimeScript RT reagent Kit (TaKaRa, Beijing, China). Evaluation of CD44 mRNA level was performed by using SYBR Premix Ex Taq Kits (TaKaRa, Beijing, China). The primer sequences of CD44 (forward:5′-AGTCACAGACCTGCCCAATGCCTTT-3′ and reverse:5′-TTTGCTCCACCTTCTTGACTCCCATG-3′) and GAPDH (forward: 5′-GACAGTCAGCCGCATCTTCT-3′ and reverse: 5′-TTAAAAGCAGCCCTGGTGAC-3′) were used and then quantified using real-time PCR on ViiA™7 Real-Time PCR System (Life Technologies, CA, USA). For the 2 − ΔΔCt method was used to evaluate the gene expression fold change among the groups. Three independent experiments were performed.

### Western blot analysis

Cells or exosomes were lysed using a radioimmunoprecipitation assay (RIPA) lysis buffer (Beyotime, Shanghai, China) supplemented with PMSF inhibitor (Beyotime, Shanghai, China). Protein lysates were loaded and separated on a 10% sodium dodecyl sulfate polyacrylamide gel and transferred onto 0.22-μm polyvinylidene fluoride (PVDF) membranes (Bio-Rad, Hercules, California, USA). The membranes were then blocked with Tris buffered saline Tween 20 (TBST) containing 5% non-fat milk for 1 h, and probed with primary antibodies overnight at 4 °C. They were then washed three times with TBST for 10 min each and probed with secondary antibodies for 1 h, followed by washing three times in TBST for 10 min per wash. The bands were visualized using an enhanced chemiluminescence (ECL) kit (Thermo Fisher, Waltham, MA, USA) in Image quant LAS400 mini (GE Healthcare, Munich, Germany). Primary antibodies against CD44 (Abclonal, Wuhan China), CD63, CD9, CD81, HSP70 (SBI, CA, USA) were used. Actin and GAPDH (Abcam, Cambridge, MA, USA) was the loading control.

### Statistical analysis

All data were tested by *F*-test and expressed as mean ± SEM. Differences were analyzed using two-tailed Student’s t-tests to perform a statistical comparison between two groups or using one-way ANOVA among three groups. Statistical tests were performed using the SPSS software, version 20.0 (SPSS Inc.) or with GraphPad Prism 6.0 (GraphPad Software, Inc.). The level of statistical significance was set at *p < 0.05, **p < 0.01, ***p < 0.001, **** *p* < 0.0001.

## Results

### Identification of exosomes from human ovarian cancer cell lines HO8910 and HO8910PM

**Fig. 1:**
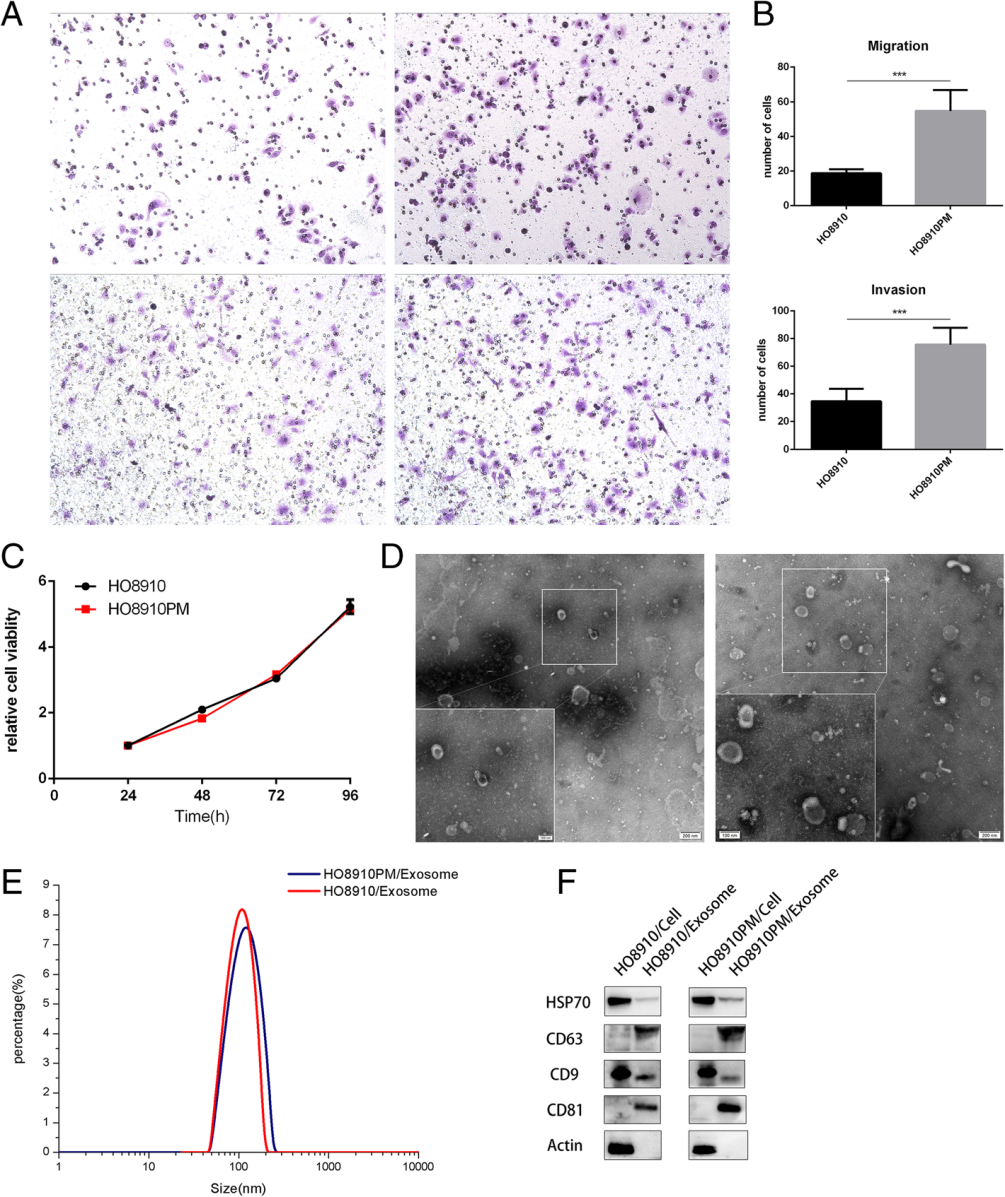
Identification of exosomes from human ovarian cancer cell line HO8910 and HO8910PM. **a** and **b** migration and invasion assay. 2 × 10^5^ and 3 × 10^5^ of HO8910 and HO8910PM cells were respectively added to trans-well plates without or with Matrigel-coated, 24 h later, migration and invasion were assessed by trans-well assays. Results in A were representative of three experiments with similar results. Results in B were shown as means ± SEM for three separate experiments. *** *p* < 0.001. **c** Cell proliferation assay. 4 × 10^3^ of HO8910 and HO8910PM cells were plated onto 96-well plates. Cell proliferation in 24 h, 48 h,72 h,96 h was respectively assessed by CCK8 assays. Results were shown as means ± SEM for three separate experiments. **d** Transmission electron microscopy. Transmission electron micrographs of purified exosomes secreted from HO8910 and HO8910PM cell lines. Scale bar, 200 nm and 100 nm. **e** Nanoparticle analysis. Concentration and size distribution of nano-sized particles in exosome suspension were measured using Litesizer 500. **f** Western blot analysis. Exosomes, isolated from HO8910 and HO8910PM cell lines, and parental cellular lysates (10 μg/lane) were western blotted for HSP70, CD63, CD9 and CD81.Actin served as the control

Human ovarian cancer cell line HO8910PM was regarded to have stronger invasive and metastatic potential than its parental cell line HO8910. We determined cellular migration and invasion ability of two cell lines using trans-well plate assays and verified that the numbers of migrated and invaded HO8910PM cells were significantly more than those of HO8910 cells (both p<0.001), as shown in Fig. 1a and b. But CCK8 assay showed that cellular proliferation ability was similar between two cells within 96 h (Fig. 1c).

Next, we extracted and identified HO8910PM and HO8910 cell-derived exosomes (PMExos and HOExos). As shown in Fig. 1d, electron microscopy revealed that both PMExos and HOExos had a round or cup-shaped morphology, with the diameter ranging 50-250 nm (median 100 nm) for PMExos and 50-200 nm (median100nm) for HOExos, respectively (Fig. 1e). Furthermore, both exosomes were enriched for exosome markers, including CD63, CD81, CD9 and HSP70 (Fig. 1f), suggesting that both cultured HO8910 and HO8910PM cells possess the ability to release exosomes.

### Exosomes derived from HO8910PM cells enhance aggressive phenotypes of HO8910 cells

**Fig. 2:**
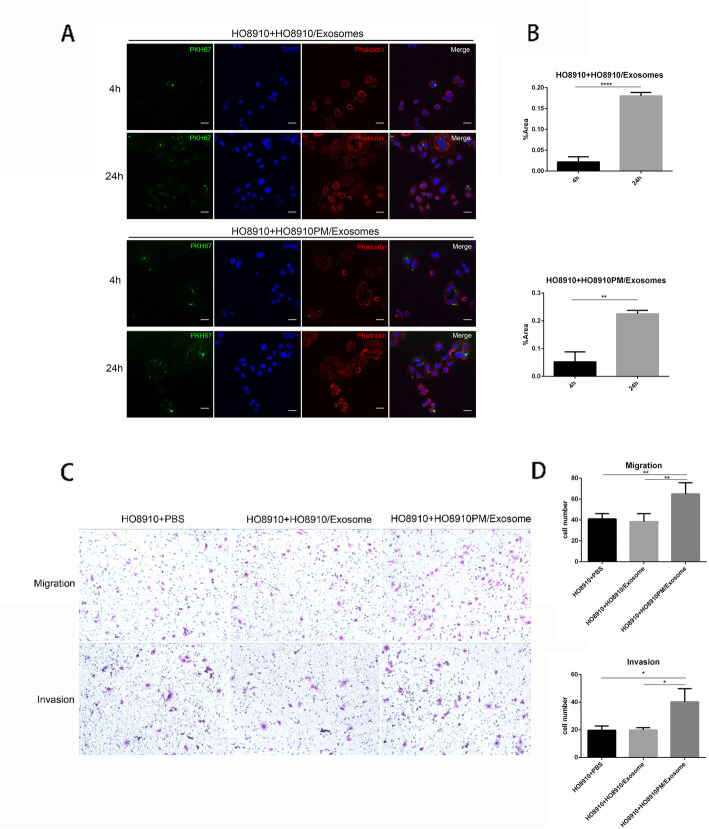
Exosomes derived from HO8910PM cells enhance aggressive phenotypes of HO8910 cells. **a**-**b** Confocal microscopy. 5 × 10^4^ HO8910 cells were cultured with exosomes labeled green fluorescent dye PKH67 for 4 h or 24 h. HO8910 cells were observed under confocal microscopy at 600 × magnification. DAPI was used to stain the nuclei and Palloidin was used to stain the cell cortex. Scale bar 20 μm**. b** Area fraction (%Area) reflected the degree of PKH67 labeled exosomes uptaking by recipient cells. Data represented the means ± SEM of three independent experiments. ****p < 0.0001, *p < 0.05 compared to 0 h. **c-d** Migration and invasion assays.10μg/mL of exosomes extracted from HO8910PM and HO8910 cells were incubated with HO8910 cells in 6-well plates for 48 h. PBS served as the control. 2 × 10^5^ and 3 × 10^5^ digested HO8910 cells were respectively added to trans-well plates without or with Matrigel-coated. Migration and invasion were assessed by trans-well assays after 24 h. Results in B were representative of three experiments. Results in D were shown as means ± SEM for three separate experiments. One-way ANOVA was used to analyze the differences. **p < 0.01, *p < 0.05

To explore whether exosomes could be transferred between cells, 10μg/ml of both PMExos and HOExos were labeled with PKH67 dye and then added into medium of cultured 5 × 10^4^ of HO8910 cells. Confocal microscopy showed that green fluorescent signals appeared in HO8910 cells after 4 h of incubation, and obviously increased after overnight incubation at 37 °C, suggesting that HO8910 cells take in both PMExos and HOExos (Fig. 2a-b).

We next investigated the effect of ingested PMExos on the phenotypes of recipient HO8910 cells. HO8910 cells were treated with PBS, 10μg/ml of HOExos and 10μg/ml of PMExos, respectively. As shown in Fig. 2c-d, HO8910 cells treated with PMExos presented increased migration and invasion ability compared to negative controls (*p*<0.01 for migration, *p*<0.05 for invasion) and PBS(p<0.01 for migration, p<0.05 for invasion), but HO8910 cells treated with HOExos did not change migration and invasion ability. Our results suggest that the stronger aggressive potential of HO8910PM cells can be transmitted to HO8910 cells via transferring exosomes, consequently enhancing metastatic potential of recipient cells.

### Screen and identification of molecules in donor exosomes affecting phenotypes of recipient cells

**Fig. 3:**
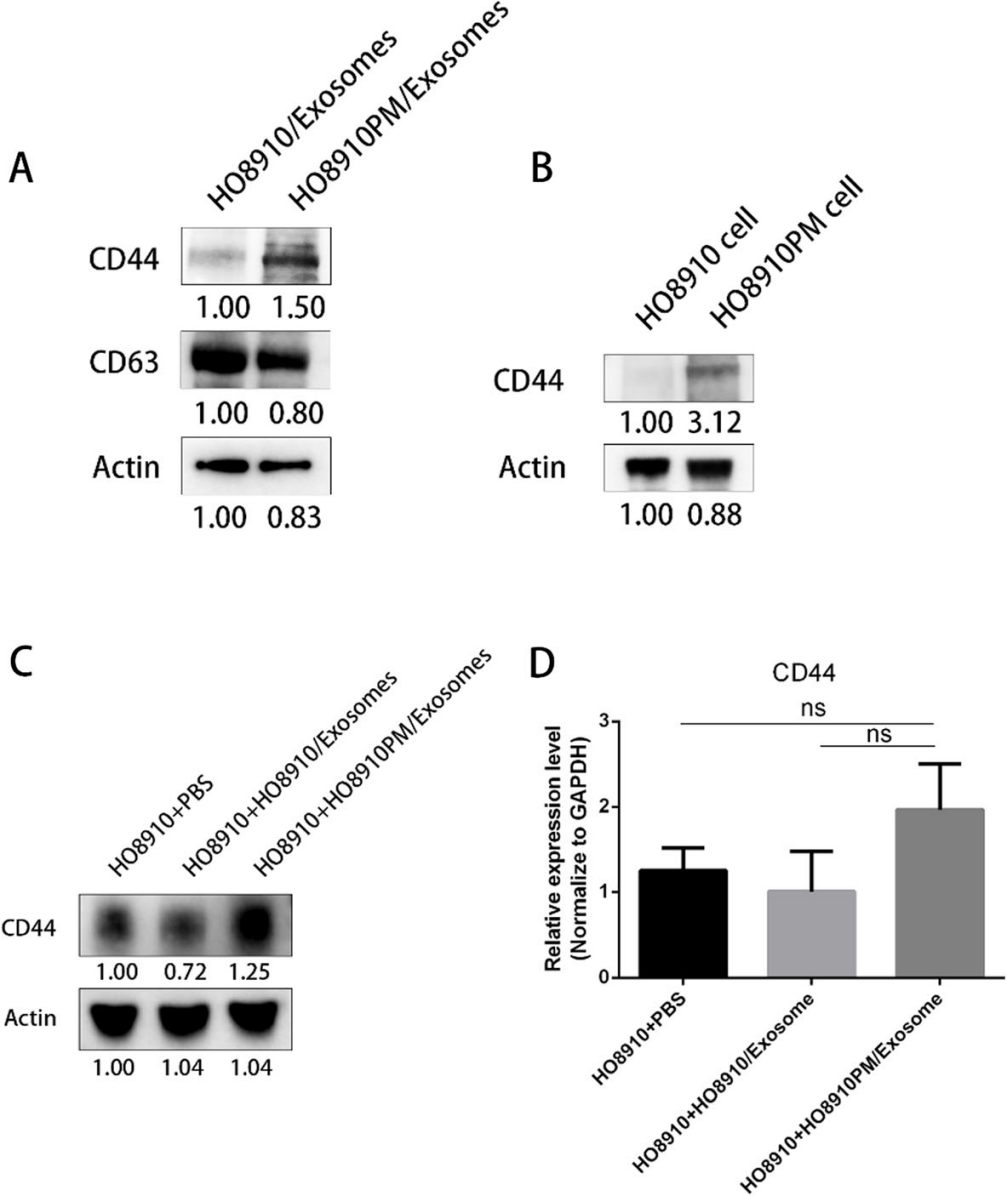
Identification of molecules in donor exosomes affecting phenotypes of recipient cells. **a** Western blot analysis on expression levels of CD44 in HOExos and PMExos (10 μg/lane). Both CD63 and Actin were used as internal controls. The blots shown were representative of three separate experiments. **b** Western blot analysis on expression levels of CD44 in HO8910 and HO8910PM cells (10 μg/lane). The blots shown were representative of three separate experiments. **c** 10μg/ml of exosomes extracted from HO8910PM and HO8910 cells were incubated with HO8910 cells in 6-well plates for 48 h. Adding PBS served as control. Thereafter, HO8910 cell lysates were collected, lysed and immunoblotting were performed (10 μg/lane). Actin was used as the internal control. The blots shown were representative of three separate experiments. **d** Real-time qRT-PCR. 10μg/ml of exosomes extracted from HO8910PM and HO8910 cells were incubated with HO8910 cells in 6-well plates for 48 h. PBS served as the control. Extracted RNA of HO8910 cells was used for qRT-PCR analysis. CD44 mRNA was detected by RT-PCR. GAPDH was used as the internal control. Results were shown as means ± SEM for three separate experiments. NS meant no significant difference

To identify the molecules in exosomes from donor cells that play a role on the phenotypes of recipient cells, we performed mass spectrometry of HOExos and PMExos. Totally 23 proteins were present in all three biological replicate MS runs. Of those, 8 proteins were always expressed higher in PMExos than that in HOExos in three biological repeats. Reversely, 4 proteins were always expressed lower in PMExos. The function of these proteins was listed as Table 1. CD44 was selected for further study because it was reported to act as adhesive molecules in cell migration. As shown in Fig. 3a, CD44 showed higher expression level in PMExos than that in HOExos, when both CD63 and actin were used as internal controls. Consistently, CD44 was also higher in HO8910PM cells than that in HO8910 cells (Fig. 3b). Next, we found that CD44 protein level in HO8910 cells treated with PMExos was significantly increased compared to cells treated with HOExos and PBS, as shown in Fig. 3c. Contrarily, mRNA level of CD44 was not changed among cells treated with PMExos, HOExos and PBS (Fig. 3d), suggesting that elevated CD44 in HO8910 cells was derived from ingested PMExos, but not synthesized in recipient cells.

**Table 1:** The function of proteins expressed differentially between PMExos and HOExos

Gene name of proteins	The expression level in PMExos	Fold change	Function
ATP1A1	High	2.394	This is the catalytic component of the active enzyme, which catalyzes the hydrolysis of ATP coupled with the exchange of sodium and potassium ions across the plasma membrane. This action creates the electrochemical gradient of sodium and potassium ions, providing the energy for active transport of various nutrients [25, 49].
TUBB	High	3.368	Tubulin is the major constituent of microtubules. It binds two moles of GTP, one at an exchangeable site on the beta chain and one at a non-exchangeable site on the alpha chain [15, 35, 43].
EEF2	High	21.891	Catalyzes the GTP-dependent ribosomal translocation step during translation elongation [18, 19].
CD44	High	1.877	Cell-surface receptor that plays a role in cell-cell interactions, cell adhesion and migration, helping them to sense and respond to changes in the tissue microenvironment [29, 31, 38, 45].
RAB6B	High	2.939	have a role in retrograde membrane traffic at the level of the Golgi complex and retrograde transport in neuronal cells [13, 44].
MSN	High	1.506	Ezrin-radixin-moesin family protein that connects the actin cytoskeleton to the plasma membrane and thereby regulates the structure and function of specific domains of the cell cortex [14, 22].
RAN	High	2.893	GTPase involved in nucleocytoplasmic transport, participating both to the import and the export from the nucleus of proteins and RNAs [34].
ACTG	High	1.147	Actins are highly conserved proteins that are involved in various types of cell motility and in maintenance of the cytoskeleton [8].
H2BFC	Low	0.267	Core component of nucleosome [1, 2].
H2AFV	Low	0.208	Variant histone H2A which replaces conventional H2A in a subset of nucleosomes [10, 40].
NID1	Low	0.615	Sulfated glycoprotein widely distributed in basement membranes and tightly associated with laminin. Also binds to collagen IV and perlecan. It probably has a role in cell-extracellular matrix interactions [4, 12, 50].
CLTC	Low	0.879	Clathrin is the major protein of the polyhedral coat of coated pits and vesicles [16].

### Exosomal CD44 is a functional mediator in intercellular communication

**Fig. 4:**
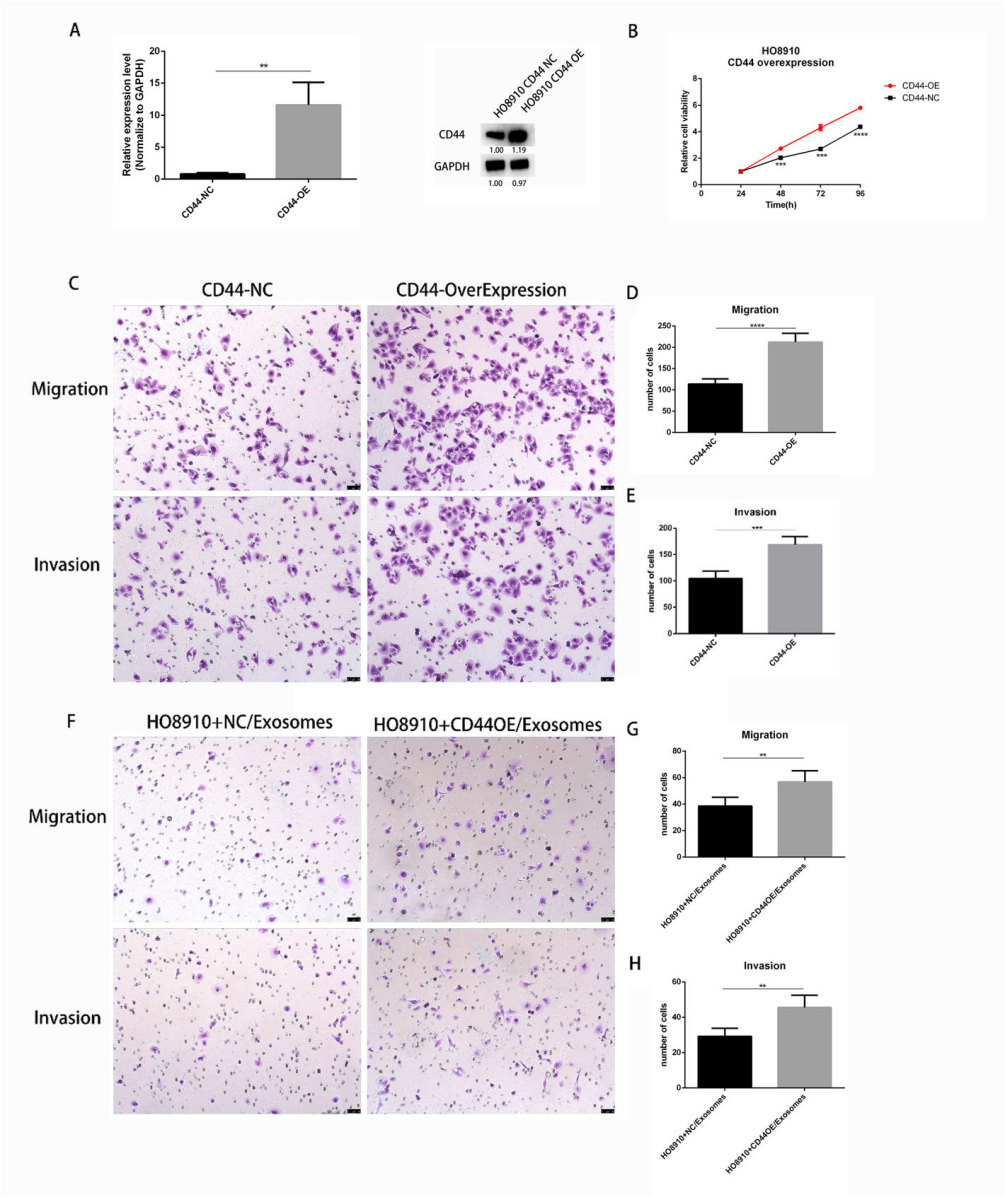
Exosomal CD44 is a functional mediator in intercellular communication. **a** Real-time qRT-PCR and Western blotting. qRT-PCR of CD44 mRNA expression after transfecting with CD44 overexpression plasmid for 24 h and treating with 500μg/ml of G418 for 2 weeks. Cell transfected with the empty vectors acted as controls. Results were shown as means ± SEM for three separate experiments, ** *p* < 0.01. Western blot analysis on cells mentioned above to validate the protein level of CD44 (10 μg/lane). The blots shown were representative of three separate experiments. **b** Cell proliferation assay. 4 × 10^3^ HO8910 cells transfected with CD44 overexpression plasmids and with empty vectors were added to 96-well plates. 24 h, 48 h, 72 h, 96 h of cell viability were respectively assessed by CCK8 assays. Results in B were shown as means ± SEM for three separate experiments,*** *p* < 0.001,****p < 0.0001. **c-h** migration and invasion assay. 2 × 10^5^ HO8910 cells transfected with CD44 overexpression plasmids and with empty vectors were respectively added to trans-well plates, and 3 × 10^5^ cells mentioned above were added to with trans-well plates with matrigel-coated.24 h later, migration and invasion were assessed by trans-well assays. Results in C and F were representative of three experiments. Results in D, E, G, H were shown as means ± SEM for three separate experiments, **p < 0.01, *** *p* < 0.001, **** *p* < 0.0001

To further verify the role of CD44 in exosomes from donor cells in changing the phenotypes of recipient cells, we overexpressed CD44 in HO8910 cells through CD44 plasmid transfection (Fig. 4a), and found that HO8910 cells stably transfected with CD44 plasmids presented increased the ability of proliferation, migration (p<0.0001) and invasion (*p*<0.001) compared to controls (Fig. 4b-e). We then extracted exosomes from HO8910 cells transfected with CD44 plasmids and empty vectors respectively, and incubated them with the HO8910 cells for 48 h. The results revealed that exosomes from HO8910 cells transfected with CD44 plasmids promoted the ability of migration (*p*<0.01) and invasion (*p*<0.01), in HO8910 cells, compared to empty vectors (Fig. 4f), suggesting that exosomal CD44 is a functional mediator in intercellular communication, and CD44 in exosomes from donor cells changes the phenotypes of recipient cells.

## Discussion

At present, a variety of methods for isolation and purification of exosomes have been developed [36], such as ultracentrifugation, gradient ultracentrifugation, co-precipitation, and others, in which ultracentrifugation-based exosome isolation is most commonly used. In our study, exosomes were successfully isolated from HO8910 and HO8910PM cells by the ultracentrifugation method. As it is hard to completely eliminate contamination pulled down from other sources in the culture medium, it is essential to identify exosomes using other approaches, including electron microscopy, particle tracking and western blotting [41]. Electron microscopy and particle tracking analysis revealed that both PMExos and HOExos had a round or cup-shaped morphology, with the diameter ranging 50-250 nm for PMExos and 50-200 nm for HOExos, respectively. Our results showed that the morphology and size were both fit to the characterization of exosomes, which were consistent with most of previous studies. As for protein markers, different cell types may have their own exosomal markers. According to the study from Théry et al., major histocompatibility complex (MHC), flotillin, and heat shock 70-kDa proteins (HSP70) were found to similarly present in all EVs. Exosomes can be distinguished by immuno-isolation using either CD63, CD81, or CD9 [21]. Thus, we tried HSP70, CD9, CD63 and CD81 and found that CD81 and CD63 were only enriched in HOExos and PMExos whereas CD9 and HSP70 also existed in cell lysates. Actin were served as internal control in this experiment. However, the expression level of actin was not detected in HO8910 and HO8910PM exosomes, which may be due to the huge expression difference of actin between cells and exosomes. In addition, we also noticed that the cell proliferation rate was similar between HO8910 and HO8910PM cells within 96 h. It is likely that the difference of cell proliferation indexes between these two cell lines could be detected when culturing for an extended period of time.

It has been well-known that exosomes act as message passengers in tumor microenvironment, and exosome transport is believed to be an effective way to modulate cell signaling or biological behavior in recipient cells [48]. The nature of tumor heterogeneity has profound implications for tumor development and therapeutic outcomes. So, exosomes may take part in the progress of tumor heterogeneity and influence the metastasis and chemotherapy resistance of tumor. Previous studies on breast cancer, renal carcinoma and ovarian cancer have shown that exosomes secreted from resistant cancer cells can promote the chemoresistance of sensitive cancer cells [7, 27, 32, 37, 46, 51]. Besides the chemoresistance phenotype, exosomes can transfer the metastasis phenotype between distinct clones. For example, it has been reported that exosomes secreted by highly metastatic clonal variants of carcinomas induce metastatic behavior in poorly metastatic clones in several cancers, including colon cancer, osteosarcoma and pancreatic ductal adenocarcinoma [23, 26, 39]. However, it is little known in ovarian cancer. We used a pair of ovarian cancer cell lines with different ability of metastasis, HO8910 and HO8910PM, and found that exosomes from HO8910PM cells contributed to the increase in migration and invasion of HO8910 cells, indicating the fact that exosomes can transfer the metastasis phenotype between distinct clones, and the extensive metastatic ability of ovarian cancer are likely to be formed by the transfer of high metastatic capacity between tumor cells. Our findings may provide a new approach to block the metastasis of ovarian cancer. However, it was worth mentioning that the human ovarian cancer cell lines which were similar to characteristics of HO8910 and HO8910PMs are rare, so further experiments in vivo were needed to verify this idea.

The surface of exosomes was decorated by the parent cell-derived signaling molecules and their intravesicular contents, including DNA, mRNA, microRNA, as well as enzymes and soluble factors, all biologically active and capable of executing functional responses in target cells and reprogramming activities of these cells [33]. For example, previous research proved that TGFβ1 in fibroblasts-derived exosomes promoted epithelial mesenchymal transition of ovarian cancer cells [24]. Besides proteins, many non-coding RNAs have been reported to be transferred through exosomes. Au yeung found that exosomal transfer of stroma-derived miR21 conferred paclitaxel resistance in ovarian cancer cells through targeting APAF 1[3]. Qu showed that exosome-transmitted lncARSR promoted sunitinib resistance in renal cancer [32]. In order to explore the factors inside exosomes that play a role in this process, we using MS analysis and Western blot confirmed that CD44 showed a higher expression in HO8910PM cells and their secreted exosomes. Because there are no consensus of internal control in exosomes, CD63, actin and GAPDH all are reported to be used as internal control [29], so in our study, we used both CD63 and Actin as internal controls to compare the expression level of CD44 between PMExos and HOExos. It has been reported that CD44 plays a role in cell-cell interactions, cell adhesion, migration and acts as a metastasis-promoting molecule in many types of cancer [29, 31, 38, 45]. As we expected, overexpression of CD44 promoted the migration, invasion and proliferation of HO8910 cells. Interestingly, CD44 protein levels were increased in HO8910 cells upon the uptake of HO8910PM-derived exosomes. Exosomes derived from HO8910 cells transfected with CD44 plasmids promoted cell migration and invasion of HO8910 cells. Our findings suggest that exosome-mediated transfer of CD44 acts as a promoter of metastatic behavior in the recipient cells.

## Conclusion

In summary, we demonstrate that exosomes secreted from highly metastatic HO8910PM cells can promote migration and invasion of lower metastatic HO8910 cells through the transfer of CD44, which suggest that the more aggressive subpopulation can transfer a metastatic phenotype to the less one via secreting exosomes within a heterogeneous tumor. Our findings may provide a potential therapeutic approach for ovarian cancer.

## Data Availability

Not applicable.
